# Intralesional Bleomycin for the Treatment of Resistant Palmoplantar and Periungual Warts

**DOI:** 10.1155/2021/8655004

**Published:** 2021-10-18

**Authors:** Suchana Marahatta, Dhan Keshar Khadka, Sudha Agrawal, Arpana Rijal

**Affiliations:** Department of Dermatology & Venereology, B. P. Koirala Institute of Health Sciences, Dharan, Nepal

## Abstract

**Introduction:**

Periungual, palmar, and plantar warts are difficult to treat with poor treatment response. Intralesional (IL) bleomycin has shown promising results for their treatment in a few reports. However, we need further evidence before opting it for treating difficult sites and resistant warts. Hence, we conducted this study to assess the efficacy and safety of IL bleomycin for the treatment of resistant palmoplantar and periungual warts.

**Methods:**

In this retrospective study, we included all patients who were given IL bleomycin for warts over a year. Maximum three sittings of bleomycin (1 mg/ml) were given monthly, and they were followed up for 3 months after the procedure. The response was categorized as complete, near-complete, significant, moderate, mild, and no clearance for 100%, 75–99%, 50–74%, 25–49%, 1–25%, and 0% clearance, respectively.

**Results:**

Out of 29 patients, follow-up details were available only in 19 patients (53 warts). The mean duration was 2.5 ± 1.47 years. The number of past interventions ranged from 2–4. Wart clearance after the first intervention was complete in 36.84%, near-complete in 26.31%, significant in 26.31%, and moderate in 10.53%. Wart clearance after the last intervention was complete in 89.47% and near-complete in 10.52% of patients. However, during 3 months of follow-up after the last injection, 15.78% had a recurrence. None of them had severe local and systemic side effects.

**Conclusions:**

IL bleomycin could be a better treatment option for the treatment of resistant and difficult warts. However, we observed a higher recurrence rate even in a shorter follow-up. Hence, we need further studies with larger samples.

## 1. Introduction

Warts are a widespread cutaneous infection which affects trauma-prone sites such as periungual areas, palms, and soles [1]. It could be more prevalent among Nepalese as most of them are farmers, walk barefoot, and share community bathing and common water sources. Although spontaneous clearance is possible, many opt for treatment for cosmetic concerns, social stigma, pain, risk of malignancies, etc. [2]. Bleomycin is an antitumor and antiviral agent commonly used for the treatment of squamous cell carcinoma [3].

Available studies have recommended cryotherapy and salicylic acid (SA) as first-line therapies for warts [4]. However, cryotherapy may not be easily available even in developing countries. SA needs a longer treatment, and the patient's compliance cannot be confirmed [1]. Moreover, a significant numbers of periungual (72%) and palmoplantar (31%) warts are resistant to common treatments. Thus, we need to be wiser while choosing the most appropriate therapeutic modalities [5].

Currently, there is limited evidence of intralesional (IL) bleomycin for treating recalcitrant warts. Some reports have shown positive results with cure rates ranging from 14%–99% [6, 7]. Hence, we conducted this study to assess the efficacy and safety of IL bleomycin for the treatment of palmoplantar and periungual warts.

## 2. Methods

In this retrospective case series, we extracted data from the eligible patient's record maintained in the operation theater (OT) of the dermatology department of BPKIHS from March 2017 to February 2018. We included patients of age ≥18 years with recalcitrant and difficult site warts, who had never received IL bleomycin in past. However, whose follow-up details were not available till three months after the last intervention and with immunocompromised status were excluded from the study. We extracted relevant patients' particulars such as age, gender, occupation, and education. We also noted clinical details such as disease duration, progression, and the name and the number of past interventions. Photographic records were maintained during all visits. Similarly, the details of examination findings such as the number, site, size of warts, and side effects were recorded at baseline as well as at each follow-up visit to assess the size reduction and efficacy of the treatment.

### 2.1. Case Definitions

  Resistant warts: they were defined as warts that have failed treatment twice in the past
  Difficult site warts: they were defined as periungual, palmar, and plantar warts

### 2.2. Injection Technique

Bleomycin for injection was obtained in vials containing 15 mg (15 U) of powder. It was first reconstituted with 5 ml sterile water for injection to prepare the stock solution, which was stored at 4–8°C for a maximum of 60 days. Then, one part of bleomycin stock solution and two parts of 2% lignocaine were taken in a 26G insulin syringe to make a final concentration of 1 U/mL (=1 mg/mL) just before injection. Each wart and the adjacent skin were cleaned with isopropyl alcohol before injection. The injection was given at the base of each wart until the lesion was blanched. The amount of injection was decided depending on the size of the warts: warts up to 5 mm, 10 mm, and more than 10 mm received 0.2 mL, 0.5 mL, and 1.0 mL of bleomycin, respectively. The total volume injected at one treatment session was limited to 2 mL, and the injection into a single wart was limited to 1 mL. Similarly, a maximum of 5 warts were treated in a session to avoid systemic side effects [8, 9]. The treated warts were given a specific number to avoid confusion during follow-up.

### 2.3. Assessment and Follow-Up

Patients were reevaluated at 4 weeks' interval. Any remaining keratotic tissues were pared off with a scalpel blade. If warts persisted or recurred after injection, the treatment was repeated using the same concentration and technique. However, it was not repeated for more than two follow-up sessions. The side effects of the injection such as pain, edema, oozing, crusting, and Raynaud's phenomenon were recorded in each follow-up visit. Patients were followed up to 3 months of the last intervention. The response was categorized as complete clearance, near-complete clearance, significant clearance, moderate clearance, mild clearance, and no clearance for 100%, 75–99%, 50–74%, 25–49%, 1–25%, and 0% clearance, respectively.

## 3. Results

Out of 29 patients (94 warts) subjected to IL bleomycin injection, only 19 subjects (=53 warts) fulfilled the inclusion criteria. The mean age of the patients was 27.95±/−11.72 years. The mean duration of the wart was 2.5±/−1.47 years, and the mean number of warts was 2.79±/−1.81 (range: 1–6). The number of past interventions ranged from 2–5. Sites of the warts were plantar = 10, periungual = 7, palmar = 1, and palmoplantar = 1 (Table 1).

**Figure 1:**
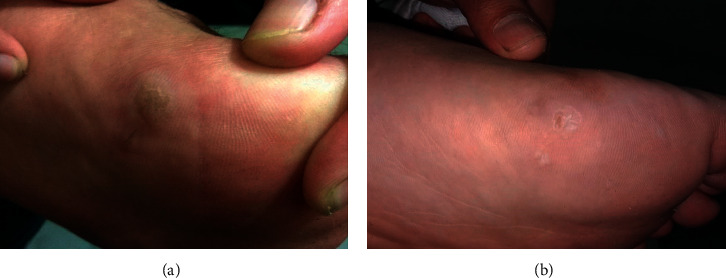
Plantar wart during baseline (a) and after one treatment session (b).

**Figure 2:**
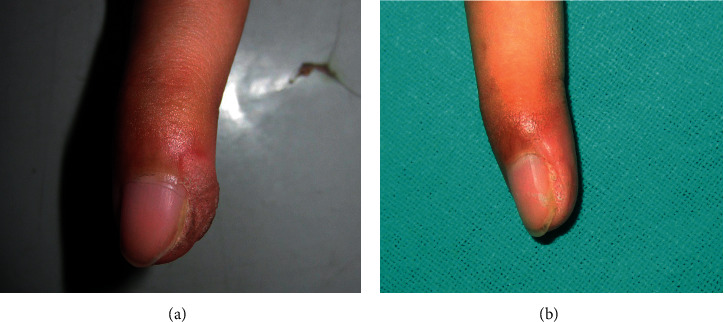
Periungual wart during baseline (a) and after two treatment sessions (b).

**Figure 3:**
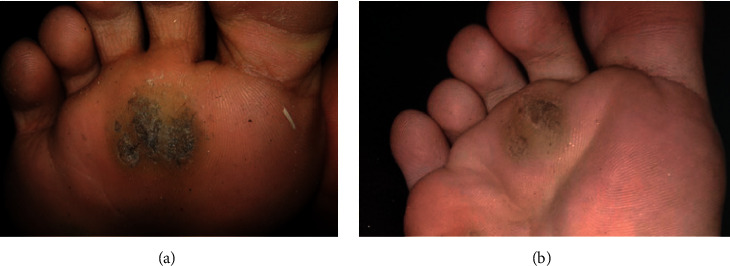
Plantar wart at baseline (a) and moderate clearance (b) after two treatment sessions.

**Figure 4:**
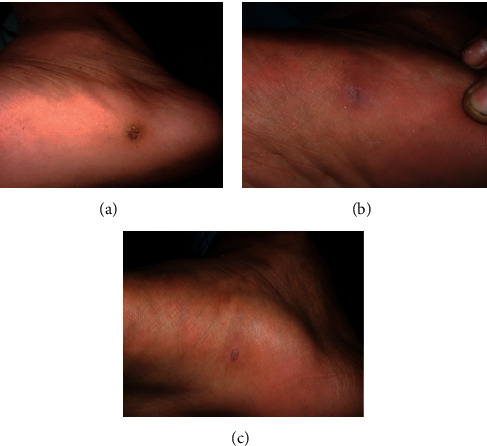
Plantar wart at baseline (a), complete clearance after one treatment session (b), and recurrence at 3 months posttreatment follow-up (c).

Wart clearance after the first intervention was complete in 7 (36.84%), near-complete in 5 (26.31%), significant in 5 (26.31%), and moderate in 2 (10.53%). Wart clearance after the last intervention was complete in 17 (89.47%) and near-complete in 2 (10.52%) patients (Figures 1–3). However, during the 3 months' follow-up after the last injection, 3 patients, i.e., 3/19 (15.78%), had a recurrence (Figure 4). None of the patients had severe local and systemic side effects. Local pain, edema, and crusting were the most common side effects and were seen in almost all patients. Duration of pain was 3 days (7 patients), 2 days (4 patients), 1 day (5 patients), and 7 days (2 patients). In three patients, we noticed temporary skin pigmentation. However, severe side effects were not seen in any of them.

## 4. Discussion

The mean duration of wart was 2.5 ± 1.47 years, and the mean number was 2.79 ± 1.81 (range: 1–6). The number of past interventions ranged from 2–4. In our study, IL bleomycin (1 U/ml) had 36.84% and 89.47% complete clearance rates after the 1st and 3rd interventions, respectively, for the treatment of difficult site recalcitrant warts. We found 15.78% recurrence rate within 3 months of the last intervention. However, it was devoid of serious adverse effects with transient pain up to 3 days being the most common (84.21%) adverse effects.

Warts are the most common cutaneous viral infection. Although self-clearance is possible in many lesions, many patients want its treatment [2]. To date, there is no specific guideline for treating nongenital warts. However, a meta-analysis and pooled analysis of RCT found the highest (58%) cure rate of the wart with cryotherapy and SA combination treatment, which were followed by aggressive cryotherapy (54%), cryotherapy (49%), SA (52%), and placebo (23%). Available evidence supports and recommends only aggressive cryotherapy and SA strongly for the treatment of warts, while higher-quality evidence is lacking to support other modalities. Treatment of recalcitrant warts at difficult sites is even more challenging. Almost 72% of periungual warts and 31% of palmoplantar warts are resistant to commonly used therapeutic modalities [5]. Moreover, cryotherapy may not be available in all centers in the developing world, and compliance cannot be insured in SA.

Bleomycin is an antitumor agent with the antiviral property. A few countable researchers had tried IL bleomycin for treating warts with inconsistent efficacy ranging from 14–99%. However, some recent reports have shown very promising results [7, 8, 10–12]. We had almost comparable complete clearance rate with the previous reports, ranging from 69.3% to 96.47% (Table 2) [8, 9, 11–16]. A little higher clearance was obtained by Dhar et al. [8] (CC: 94.9%), including all cutaneous warts without prior treatment. Soni et al. (CC: 96.47%) had selected difficult site warts, but they had also not received any treatment in the past [9]. However, we had chosen difficult site warts with treatment failure with at least 2 prior treatment sessions with other modalities.

Alghamdi et al. [17] treated most of the periungual warts with past treatment failure with much lesser bleomycin concentration using the multipuncture technique, with good clearance rate (86.6%) and lesser recurrence rate (13.33%) [17]. Unlike other reports, we had a higher recurrence rate (15.78%). The possible explanation could be that we have not conducted a dermoscopic evaluation to find complete clearance. As supported by the work of Barkat et al., without dermoscopic evaluation, we may miss 19.2% of cases. In their study, 69.3% clearance was obtained with dermoscopy evaluation, but 88.5% clearance was obtained when evaluated only clinically [12]. In the work of Dhar et al., recurrence was lesser (13%) than ours. However, they had chosen all patients without prior treatment and the follow-up duration was 4 weeks lesser than that of our study [8]. But, we had selected only recalcitrant warts with a slightly longer follow-up. Hence, we might have a higher recurrence. However, in a study that has chosen recalcitrant plantar warts, 19.35% recurrence was seen in 6 months [16]. Similarly, in a study which included difficult site recalcitrant warts, RR was 23% in 6 months (Table 2) [5].

Pain for a few initial days was the most common side effect; transient pigmentation was seen among 3 patients. However, none of them had serious adverse effects. These findings are comparable to other reports [9, 12–18]. One previous study detected reversible necrosis in 1.2% of the patients with IL bleomycin injected for ungual warts [19]. However, we did not observe such complications.

### 4.1. Limitations of the Study

Retrospective case series with lesser sample size and shorter follow-up after the last intervention are the major limitations of our study.

## 5. Conclusions

In our study, there was a good number of complete clearance (89.47%) after two sessions of IL bleomycin for the treatment of difficult sites and resistant warts. None of the participants had severe adverse effects. Therefore, IL bleomycin could be a safe, better, and promising treatment option for resistant warts in difficult sites. However, we observed a higher recurrence rate even in a shorter follow-up period (15.78%). We need further studies, preferably randomized controlled trials with larger samples. Furthermore, we recommend including dermoscopic evaluation for the assessment of complete clearance.

## Figures and Tables

**Table 1:** Baseline characteristics and treatment outcome of the warts.

S. no.	Age (in years)/gender	Site of wart	Number of past interventions	No. of warts	Past procedure	Size of the largest wart (mm)	Treatment sessions	Outcome
2	37/M	Periungual	2	2	CO2 LASER	10 × 5	2	CC
3	56/F	Periungual	3	3	EC and excision	10 × 5	1	CC
4	19/F	Plantar	2	4	EC	6 × 6	2	CC
5	16/M	Plantar	2	1	Excision	15 × 15	1	CC
6	28/M	Periungual	2	1	EC	10 × 6	2	CC
7	18/F	Periungual	3	2	EC	15 × 5	2	CC
8	18/F	Plantar	4	5	Chemical cautery	25 × 15	3	R
9	24/M	Plantar	2	3	EC	6 × 6	2	CC
10	19/F	Plantar	2	5	EC	3 × 5	1	CC
11	37/M	Periungual	2	1	CO2 LASER	10 × 5	2	CC
12	19/M	Plantar	5	6	EC	25 × 20	2	CC
13	19/F	Periungual	2	1	EC	9 × 4	1	CC
14	34/F	Plantar	3	6	Excision	12 × 10	1	CC
15	21/F	Palmoplantar	3	4	Excision	10.8	2	R
16	35/F	Plantar	2	1	Chemical cautery and EC	12 × 11	3	NC
17	25/M	Palmar	3	1	EC	10 × 9	3	NC
18	26/M	Plantar	2	2	EC	12 × 10	1	CC
19	27/M	Plantar	2	4	Excision	10 × 5	2	R
20	54/F	Periungual	3	1	EC	25 × 15	1	CC

EC: electrocautery, CC: complete clearance; NC: near-complete clearance; R: recurrence.

**Table 2:** Literature review (from the year 2005 to August 2020).

Authors	Intervention (study design)	Results	Follow-up duration
Our study	IL bleomycin only (retrospective case series)	CC (89.47%), RR (15.78%)	3 months
Barkat et al. [12]	IL bleomycin vs. placebo (RCT)	Bleomycin = dermoscopic clearance (69.3%); CC (88.5%), CC of placebo (0%)	3 months
Pasquali et al. [13]	IL bleomycin + electropolation vs. IL bleomycin only (prospective case series)	CC of IL bleomycin + electroporation (78%), bleomycin only (16%)	3 months
Dhar et al. [8]	IL bleomycin vs. cryotherapy (RCT)	CC of bleomycin (94.9%); cryotherapy (76.5%); RR (13%)	8 weeks
Adalatkhah et al. [14]	IL bleomycin vs. cryotherapy (RCT)	CC of bleomycin (86%), cryotherapy (68%)	6 weeks
Al-Naggar et al. [15]	IL bleomycin vs. microneedling assisted topical bleomycin spray (RCT)	CC of IL bleomycin (70%), CC of microneedling-assisted topical spray (83.3%)	6 months
Soni et al. [9]	IL bleomycin vs. placebo (RCT)	CC of IL bleomycin (96.47%) vs. placebo (11.11%)	12 months
Salk and Douglas [16]	IL bleomycin only (prospective study)	CC (87%), RR (19.35%)	6 months
Alghamdi and Khurram [17]	Translesional multipuncture technique with 0.1 U/ml concentration for periungual warts (prospective study)	CC (86.6%), RR (13.33%)	6 months
Alghamdi and Khurram [11]	Translesional multipuncture technique with 0.1 U/ml concentration for plantar warts (prospective study)	CC (74%), no response (13%), RR: 9.5%	3 months
Aziz-Jalali et al. [5]	IL bleomycin (retrospective study)	CC (73%), RR (23%)	6 months

RCT: randomized controlled trial, CC: clinical clearance, RR: recurrence rate.

## Data Availability

Data will be deposited in a repository, or they can also be obtained from the corresponding author on request.
